# Sexual Abuse in Adolescents Is Associated With Atypically Increased Responsiveness Within Regions Implicated in Self-Referential and Emotional Processing to Approaching Animate Threats

**DOI:** 10.3389/fpsyt.2020.00345

**Published:** 2020-06-16

**Authors:** Karina S. Blair, Johannah Bashford-Largo, Niraj Shah, Jennie Lukoff, Jaimie Elowsky, Steven Vogel, Amanda Emmert, Ru Zhang, Matthew Dobbertin, Seth Pollak, James R. Blair

**Affiliations:** ^1^Center for Neurobehavioral Research, Boys Town National Research Hospital, Boys Town, NE, United States; ^2^Department of Psychiatry, Creighton University School of Medicine, Omaha, NE, United States; ^3^Department of Psychology, University of Wisconsin, Madison, WI, United States

**Keywords:** childhood sexual abuse, threat responsiveness, looming threat, adolescents, functional Magnetic Resonance Imaging (fMRI)

## Abstract

Childhood sexual abuse is associated with significant subsequent pathology and neurodevelopmental disruption. In particular, childhood sexual abuse has been associated with heightened threat sensitivity. However, little work has directly investigated this issue. In this study, we examine the association of childhood sexual abuse to neural and behavioral responses to looming, threatening face stimuli. The study involved 23 adolescents with significant past sexual abuse and 24 comparison individuals matched on IQ, age, and sex. Participants were scanned during a looming threat task that involved negative and neutral, human faces and animals that appeared to either loom toward or recede from the participant. We found that adolescents who had been previously subjected to sexual abuse, relative to comparison adolescents, showed increased neural responses to *threatening looming stimuli* in regions including rostral and superior frontal gyrus as well as posterior cingulate gyrus. In addition, they were significantly more slowed by looming stimuli, particularly if these were human faces, than adolescents who had not been exposed. These data demonstrate that prior sexual abuse was associated with heightened neural responsiveness to looming threats in a series of regions beyond the amygdala. These data are interpreted within models of rostromedial frontal and posterior cingulate cortices that stress their role in self-referential emotional processing and emotional maintenance.

## Introduction

Childhood sexual abuse is relatively common and a major risk factor in the development of psychopathology [e.g., (1–3)]. It may have a particularly adverse impact relative to other forms of maltreatment (4, 5) with epidemiological work indicating that it is the most common cause of posttraumatic stress disorder (PTSD) (6). Despite this, studies focusing on the neurodevelopmental impact of sexual abuse are relatively rare [cf. (7, 8)].

Individuals subjected to maltreatment report difficulty regulating emotional responses to adverse events (9, 10). This is associated with increased responses to threat within the amygdala (9, 11–14). However, there has been less attention with respect to maltreatment and regions beyond the amygdala though regions though reports exist of regions such as medial frontal, posterior cingulate (PCC), and superior temporal cortices showing atypical responding to threat following maltreatment (8, 11, 15, 16). While there are no definitive accounts of the roles of these regions in threat processing, cases can be made that ventromedial frontal and PCC cortices are critically involved in the representation of stimulus value [e.g., (17)] while rostromedial frontal cortex may be particularly implicated in the maintenance of emotional responses (18).

Neural responses to threat are not uniform (19). Neural responses to relatively basic threats (e.g., a stimulus looming toward the individual) show overlaps with, but also distinctions from, those to threats that are more visually complex; e.g., facial expressions and threatening animal images (e.g., a snarling wolf); see **Supplemental Figure 1**. Moreover, core neural regions implicated in the response to threats such as the amygdala also respond to other stimulus classes such as human faces (20, 21). Yet again, the differential response to the human faces vs. animal stimulus dimension is rather different from those to the looming or visual threat dimensions properties; see Coker-Appiah et al. (19) and **Supplemental Figure 1**. It is currently unclear the extent to which maltreatment, and particularly sexual abuse, exaggerates responsiveness to particular types of threat stimuli and, perhaps more critically, to social stimuli such as faces that recruit at least partially overlapping neural circuitry (e.g., the amygdala). The level of responsiveness to social stimuli is interesting for two reasons: First, heightened responsiveness to such social stimuli is associated with social anxiety (22–24) that would further detrimentally affect the lives of individuals who had suffered abuse. Second, it allows a determination of the extent to which hyperresponsiveness to threat in specific neural systems (amygdala, vmPFC) is stimulus specific or neural region specific (i.e., to threats only or to all stimuli types that these regions respond to).

The current study aimed to determine the extent to which childhood sexual abuse is associated with increased threat responsiveness to both basic threat properties of a stimulus (e.g., looming relative to receding) as well as more visually complex properties (e.g., angry relative to neutral expressions/snarling threatening animals relative to neutral animals). In addition, it sought to determine whether increased responsiveness is also seen to human faces relative to animals given the degree of overlap of neural systems engaged by both human faces and threatening stimuli (21). This was investigated with a group of participants previously subjected to sexual abuse and an age, IQ, and sex matched comparison group. Participants performed the Looming task (19). On the basis of the previous literature (9, 11–13), we predicted that participants subjected to prior sexual abuse would show increased responses to both looming relative to receding, and threatening relative to neutral images and potentially particularly strong responses to *looming threatening* images within the amygdala but also associated structures (medial frontal, anterior insula, PCC, and superior temporal gyrus). With respect to human relative to nonhuman faces, we predicted that if sexual abuse has a general impact in increasing responsiveness within regions such as the amygdala and medial frontal cortex, then individuals subjected to sexual abuse will show greater responses within these regions to human faces than comparison individuals. In contrast, if the impact of sexual abuse more selectively impacts the responsiveness of these regions to threat, we anticipated observing group differences only in response to threat variables.

## Materials and Methods

### Participants

Twenty-three adolescents who reported significant past sexual abuse and 24 adolescents who did not report past sexual abuse participated in the study. The two groups were matched on age, sex, and IQ (see **Table 1**). Past sexual abuse was indexed *via* the Childhood Trauma Questionnaire (CTQ). Following validated thresholds [e.g., (25, 26)], individuals were considered to have experienced significant past sexual abuse if they endorsed three or more items pertaining to sexual abuse on the CTQ (see below for details about the CTQ). All sexual abuse reported was officially documented and under care. Psychiatric characterization was done through psychiatric interviews by licensed and board-certified psychiatrists with the participants and their parents, to adhere closely to common clinical practice. Individuals in the healthy comparison (HC) group reported no past sexual abuse and no current/past history of psychiatric illness.

**Table 1:** Participant characteristics.

	No past abuse (N = 24)	Past sexual abuse (N = 23)
**CTQ Sexual Abuse score^**	–	16.5 (SD = 5.32); R: 8–25
**Age**	15.1 (SD = 1.87)	15.1 (SD = 1.58)
**IQ**	99.9 (SD = 10.82)	95.3 (SD = 8.57)
**% Female**^a^	63% (N = 15)	74% (N = 17)
**MDD**^b^	0% (0)	30.4% (N = 7)
**PTSD**^b^	0% (0)	43.5% (N = 9)
**Stimulants**^c^	0% (0)	17.4% (N = 4)
**Antidepressants**^c^	0% (0)	34.8% (N = 8)
**Antipsychotics**^c^	0% (0)	34.8% (N = 8)

^Sexual Abuse as indexed by the Childhood Trauma Questionnaire (CTQ) Sexual Abuse subscale; MDD, major depressive disorder; PTSD, posttraumatic stress disorder.

^a^self-identified as female; ^b^clinical diagnosis by psychiatrist; ^c^medication currently prescribed.

Participants who had experienced sexual abuse were recruited shortly after their arrival at a residential care facility. Comparison adolescents were recruited from the community. Participants were excluded if IQ was below 75 assessed with the Wechsler Abbreviated Scale of Intelligence (WASI two-subtest form) or if they had nonpsychiatric medical illnesses that required the use of medication that may have psychotropic effects, such as beta-blockers or steroids. However, medications provided for psychiatric disorders (specifically antipsychotic, stimulant, or mood stabilizing medications) were not exclusory. Exclusion criteria also included braces, claustrophobia, active substance dependence, pervasive developmental disorder, Tourette’s syndrome, lifetime history of psychosis, neurological disorder, head trauma, non-English speaking, and presence of active safety concerns.

Written informed consent and assent was taken. In all cases, youth had the right to decline participation at any time before or during the study. Consent documents were reviewed with the parent/legal guardians and written permission was obtained (1) at the initial visit for community participants or (2) at the time of intake for youth placed in Boys Town programs. Assent was obtained from the Boys Town youth in a separate session. It was made clear to all participants and their parents that their decision with respect to participation had no influence on their clinical care. The Boys Town National Research Hospital institutional review board approved this study.

### Measures

History of sexual abuse was assessed using the CTQ, a 28-item self-report measure that indexes childhood/adolescent maltreatment. The CTQ indexes sexual abuse *via* 5 items: (1) Someone tried to touch me in a sexual way/made me touch them; (2) Someone threatened me unless I did something sexual; (3) Someone tried to make me do/watch sexual things; (4) Someone molested me; and (5) I believe that I was sexually abused. In addition to the sexual abuse scale, the CTQ contains four other subscales indexing emotional abuse (EA), physical abuse (PA), emotional neglect (EN), and physical neglect (PN). The CTQ has excellent psychometric properties including internal consistency, test-retest reliability, and convergent and discriminant validity with interviews and clinician reports of maltreatment (27). Individuals respond to each item using a five-point Likers scale: (1) never true, (2) rarely true, (3) sometimes true, (4) often true, and (5) very often true. Thus, individuals can score between 5 (no history of abuse) and 25 (extreme abuse) on the sexual abuse subscale of the CTQ. Psychiatric diagnoses were made according to the Diagnostic and Statistical Manual of Mental Disorders-5 (American Psychiatric Association, 2013) through psychiatric interviews by licensed and board-certified child and adolescent psychiatrists with the participants and their parents, to adhere closely to common clinical practice.

### fMRI Task

The participants performed the looming task *(adapted from Coker-Appiah et al., 2013)*. They were presented with an image that appeared to either loom toward or recede away from them. Images were human or animal faces and were either threatening or neutral in valence.

**Figure 1:**
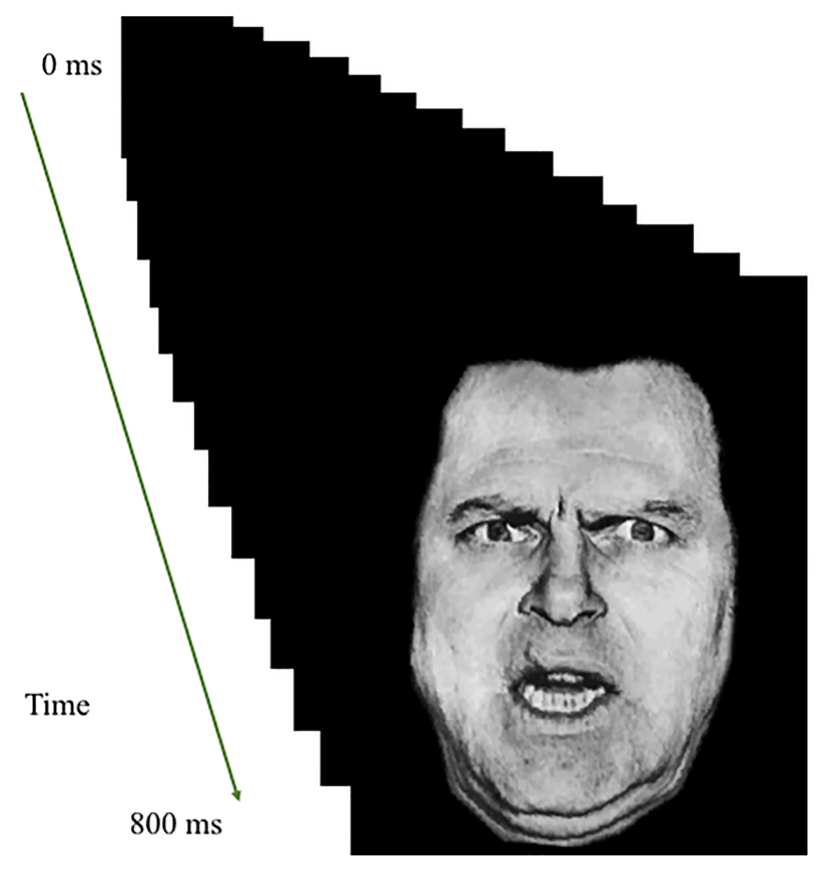
Task illustration. Example of Looming Threatening Human face trial.

Images were rapidly presented in a series of sixteen 50-ms frames of increasing or decreasing size in the center of the screen to create the effect of either looming (i.e., increasing in size in rapid succession) or receding (i.e., decreasing in size in rapid succession; total stimulus duration: 800 ms); see **Figure 1**. Stimulus presentations were followed by a fixation point, which was on screen for a jittered duration of 1,250–4,250 ms. The task included a single run of 160 stimuli (20 of each of the 8 trial types). Each individual image was presented only twice; once looming toward and once receding from the participant. In order to ensure attention to the task, participants were instructed to press a button with their right index finger as quickly as possible when an image appeared on the screen.

### fMRI Parameters

Whole-brain blood oxygen level dependent (BOLD) fMRI data were acquired using a 3.0 Tesla Siemens Skyra Magnetic Resonance Scanner. Functional images were taken with a T2* weighted gradient echo planar imaging (EPI) sequence [repetition time (TR) = 2, 500 ms, echo time (TE) = 27 ms, flip angle = 90° field-of-view (FOV) = 240 mm]. Whole-brain coverage was obtained with 43 axial slices (thickness, 2.5mm; voxel size 2.6 × 2.6 × 2.5 mm^3^; distance factor 21%). In the same session, a high-resolution T1-weighed anatomical image was acquired to aid with spatial normalization (MP-RAGE, repetition time = 2,200 ms, echo time = 2.48 ms; 230-mm field of view; 8° flip angle; 256 × 208 matrix) was acquired to register with the EPI dataset. Whole-brain coverage was obtained with 176 axial slices (thickness 1 mm; voxel size 0.9 × 0.9 × 1 mm^3^, distance factor 50%).

### fMRI Analysis: Data Preprocessing and Individual Level Analysis

Functional MRI data were preprocessed and analyzed using Analysis of Functional NeuroImages (AFNI) software (28). Data from the first four repetitions were collected prior to magnetization equilibrium and were discarded. The anatomical scan for each participant was registered to the Talairach and Tournoux atlas (29) and each participant’s functional EPI data were registered to their Talairach anatomical scan in AFNI. Functional images were motion corrected and spatially smoothed with a 6-mm full width half maximum Gaussian kernel. The data then underwent time series normalization and these results were multiplied by 100 for each voxel. Therefore, the resultant regression coefficients are representative of a percentage of signal change from the mean.

A model was generated using six motion regressors and the following eight regressors: Looming Threatening Human, Looming Neutral Human, Looming Threatening Animal, Looming Neutral Animal, Receding Threatening Human, Receding Neutral Human, Receding Threatening Animal, Receding Neutral Animal. GLM fitting was performed with these eight regressors, six motion regressors, and a regressor modeling baseline drift. All regressors were convolved with a canonical hemodynamic response function (HRF) to account for the slow hemodynamic response (with time point commencing at time of first image onset). This produced a β coefficient and associated *t* statistic for each voxel and regressor. There was no significant regressor collinearity.

### Statistical Analyses

*Behavioral Data:* A 2 (Group: Subjected to sexual abuse, Comparison) by 2 (Direction: Looming, Receding) by 2 (Type: Human, Animal) by 2 (Valence: Threatening, Neutral) ANOVA was conducted on the participant’s mean reaction times (RT) for each trial type. Outliers (RTs 3 standard deviations greater or lesser than the participant’s mean RT for that trial type) were excluded (approximately 1% of the data).

*MRI Data:* Our hypotheses were tested by a full 2 (Group: Subjected to sexual abuse, Comparison) by 2 (Direction: Looming, Receding) by 2 (Type: Human, Animal) by 2 (Valence: Threatening, Neutral) ANOVA performed on the BOLD data. Subsequently, we ran a second group-based ANCOVA where total levels of other forms of maltreatment (physical and emotional abuse and physical and emotional neglect) was the covariate. For the follow-up ANCOVAs a Blom Transformation was applied to the participants’ CTQ sexual abuse and the CTQ other maltreatment (EA + PA + EN + PN) scores. This is a normalization procedure which rank orders, and then standardizes values within a dataset (30). To facilitate future meta-analytic work, effect sizes [partial eta square (ηp²)] are reported in the Tables [though note that our relatively small sample size may result in an overestimate of the size of the true population effect; (31)].

Correction for multiple comparisons was performed using a spatial clustering operation in AFNI’s 3dClustSim utilizing the autocorrelation function (-acf) with 10,000 Monte Carlo simulations for the whole-brain analysis. Spatial autocorrelation was estimated from residuals from the individual-level GLMs. The initial threshold was set at *p* =.001. This process yielded an extant threshold of *k* = 20 voxels for the whole brain (multiple comparison corrected p < 0.05). Follow-up testing was conducted within the Statistical Package for the Social Sciences (SPSS) version 22.0.0.2 (IBM Corporation, Armonk, NY).

## Results

### Behavioral Data

A 2 (Group: Subjected to sexual abuse, Comparison) by 2 (Direction: Looming, Receding) by 2 (Type: Human, Animal) by 2 (Valence: Threatening, Neutral) ANOVA was conducted on the reaction time (RT) data. There was a main effect of Direction [F(1,45) = 68.92; p < 0.001; ηp² = 0.616]; participants were faster to respond to receding than looming stimuli (461.55 vs. 411.26 ms). In addition, there was a significant: (i) main effect of Group [F(1,45) = 9.05; p = 0.004; ηp² = 0.174; participants that had been subjected to sexual abuse were significantly slower to respond than comparison adolescents; M(subjected to sexual abuse) = 481.46 ms; M(Comparison) = 391.35 ms]; (ii) Group-by-Direction interaction [F(1,45) = 5.02; p = 0.030; ηp² = 0.104]. Participants that had been subjected to sexual abuse showed a significantly greater increase in RT for looming relative to receding stimuli relative to comparison adolescents [F(1,45) = 5.21; p = 0.030; M(subjected to sexual abuse: Looming-Receding) = 63.87ms; M(Comparison: Looming-Receding) = 36.73ms]; and (ii) a significant Group-by-Direction-by-Type interaction [F(1,45) = 7.08; p = 0.010; ηp² = 0.14]. Participants who had been subjected to sexual abuse showed significantly greater increases in RT to looming human faces than receding human faces relative to comparison adolescents {t[45]=−3.31; p=0.002; M[subjected to sexual abuse: Looming-Receding(human faces)] = 83.09ms; M[Comparison: Receding-Looming(human faces)] = 36.87ms}. However, there were no significant group differences for animal stimuli {t[45] = −0.566; p = 0.574; M[subjected to sexual abuse: Receding-Looming(animal stimuli)] = 44.64 ms; M[Comparison: Receding-Looming(animal stimuli)] = 36.59 ms}.

### Movement Data

Volumes were censored if there was >0.5 mm motion across adjacent volumes. No participant in the final sample for the current study had >5% censored volumes. There were no significant group differences in terms of censored volumes (F < 1; ns), average motion per volume (F < 1; ns)], or maximum displacement during scanning (F = 1.73; p = 0.195).

### fMRI Data

The analysis of the BOLD response data revealed regions showing significant Group-by-Direction-by-Valence interactions (see **Table 2**). All other significant results are listed in **Supplemental Table 1**. Images of the main effects of Direction, Valence and Type are presented in **Supplemental Figure 1**.

**Table 2:** Significant areas of activation from the initial 2 (Group: Subjected to sexual abuse, Comparison) by 2 (Direction: Looming, Receding) by 2 (Type: Human, Animal) by 2 (Valence: Threatening, Neutral) ANOVA.

REGION	BA	Voxels	X	Y	Z	F-value	ηp²
***Group-by-Direction-by-Valence***							
L medial frontal gyrus	10	68	−1	59	20	26.32	0.369
L superior frontal gyrus	10	29	−22	53	26	24.22	0.350
L superior frontal gyrus	8	26	−13	44	41	24.82	0.356
L posterior cingulate cortex	31	49	−1	−49	32	21.37	0.322
R superior temporal gyrus	38	31	41	14	−31	28.71	0.389
R inferior temporal gyrus	20	23	50	−7	−19	30.04	0.400

Activations are from whole brain analyses significant at p < 0.001, corrected for multiple comparisons significant at p < 0.05.

#### Group-by-Direction-by-Valence Interactions

**Figure 2:**
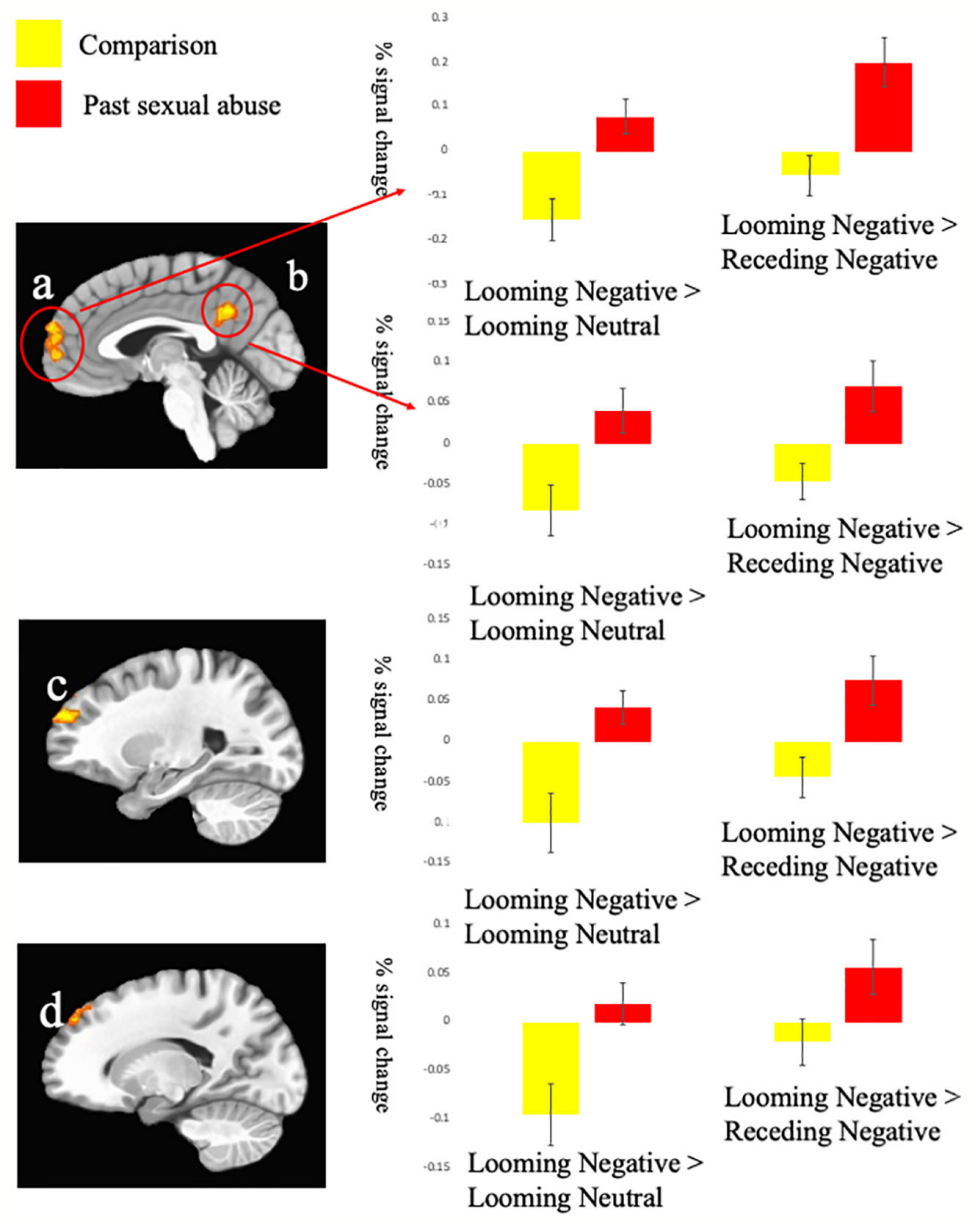
Interactions of Group-by-Direction-by-Valence. Blood oxygen level dependent (BOLD) responses within **(A)** left medial prefrontal gyrus (−1, 59, 20); **(B)** left posterior cingulate cortex (−1, −49, 32); **(C)** left superior frontal gyrus BA 10 (−22, 53, 26); and **(D)** superior frontal gyrus BA 8 (−13, 44, 41) to the Looming Negative trials compared to the Looming Neutral and Receding Neutral trials.

Regions showing significant Group-by-Direction-by-Valence interaction included rostromedial prefrontal cortex (rmPFC) (BA 10), superior frontal gyrus (BA 8 & BA 10), as well as superior (BA 38) inferior (BA 20) and superior temporal gyrus, and PCC (BA 31); **Table 2**. Within all these regions, participants who had been subjected to sexual abuse, relative to comparison individuals, showed significantly greater BOLD responses to *looming threatening* stimuli relative to both *looming neutral* (F range = 7.35 to 14.20; p < range = 0.01 to 0.001) and *receding threatening* stimuli (F range = 4.35 to 12.93; p < range 0.005 to 0.001); see **Figure 2**. A region of amygdala also showed a Group-by-Direction-by-Valence interaction but at marginal levels (p < 0.05, uncorrected).

### Follow-Up Analyses

1. ***Excluding Participants With PTSD:*** Given the potential influences of PTSD pathology (nine participants subjected to sexual abuse had diagnoses of PTSD), we reran the initial group-based analysis excluding the participants with PTSD diagnosis. Even when excluding those participants, the Group-by-Direction-by-Valence result pattern reported above and in **Table 2** largely remained; see **Supplemental Table 2**).
2. ***Excluding Participants With MDD:*** Given the potential influences of MDD pathology (seven participants subjected to sexual abuse had diagnoses of MDD), we also reran the initial group-based analysis excluding the participants with MDD diagnosis. The exclusion of those participants did not significantly change the results reported above in the main analysis and in **Table 2**; see **Supplemental Table 3**).
3. ***Excluding Participants on Medication:*** Given the potential influences of mediation use, the group-based ANOVA above was re-run excluding participants on medication (stimulants, antidepressant, and antipsychotics). Again, excluding these participants did not change the Group-by-Direction-by-Valence result pattern reported above and in **Table 2**. See **Supplemental Tables 4–6**.
4. ***Other Maltreatment as an Added Covariate to CTQ Sexual Abuse Scores:*** Given the potential overlap in neural correlates associated with other types of maltreatment, we followed up our initial ANOVA analysis with a group based ANCOVA where Blom transformed total levels of other forms of maltreatment (physical and emotional abuse and physical and emotional neglect) was the covariate. The Group-by-Direction-by-Valence interactions for the left medial, left superior frontal gyrus (BA 8) and the superior temporal gyrus remained; see **Supplemental Table 7**. In contrast, there were no interactions surviving for the Other-Maltreatment-by-Direction-by-Valence interaction.

## Discussion

In this study, we investigated the extent to which past sexual abuse is associated with increased responsiveness to threat (looming stimuli and threat images) and social cues (human faces). There were three main findings: First, participants who had been subjected to sexual abuse showed heightened neural responsiveness to looming threats in a series of regions beyond the amygdala (e.g., rmPFC and PCC). Second, participants who had been subjected to sexual abuse did not show heightened responsiveness specifically to social stimuli. Third, looming stimuli were generally associated with slower reaction times and this exaggerated in participants previously subjected to sexual abuse particularly if the looming stimulus was a human face.

Previous work has reported that participants who have experienced maltreatment show heightened responsiveness to threat (8, 11, 13–15). However, much of this work has focused on the amygdala (9, 11–14). There was a Group-by-Direction-by-Valence interaction within the amygdala in the current study. However, this was of marginal significance (p < 0.05 uncorrected). Instead, the results of the current study join other work stressing the impact of maltreatment, in this case past sexual abuse, in increasing responsiveness in regions beyond the amygdala [cf. (16, 32)]. This failure to observe more of an increase in amygdala responsiveness in participants who had been subjected to sexual abuse did *not* reflect task or imaging parameters. As reported in **Supplemental Table 1**, and seen in **Supplemental Figure 1**, the amygdala showed strong responses to looming relative to receding stimuli, threat relative to neutral images and human faces relative to animals. However, this amygdala responsiveness was not modulated by previously being subjected to sexual abuse. There was no significant Group-by-Direction, Group-by-Valence, or Group-by-Type within the amygdala even at p < 0.05. We assume that this reflects a Type II error perhaps as a result of specific features of the task/scanner but future work will investigate this further.

rmPFC, vmPFC, and PCC have been implicated in assessing the salience and relevance of emotional stimuli (17, 18, 33, 34). The suggestion has been made that the intensity of an emotional experience is due in part to the role of these cortical midline structures in affect-based self-referent processing (35, 36). In addition, it has been suggested that rmPFC is particularly implicated in the maintenance of this emotional response (18). As such, it can be speculated on the basis of the current data that being previously subjected to sexual abuse may lead to a heightened emotional response that reflects self-referential emotional processing and emotional maintenance. The amygdala may be implicated but the functioning of other structures, particularly those involved in affect-based self-referent processing and emotional maintenance, may be even more impacted (at least by being previously subjected to sexual abuse). One feature of the current results that is interesting in this regard is that the interactions of group were with Direction-by-Valence, not Direction or Valence alone. This is interesting because in the current results and similar to our previous experience with this task (19), there were no regions showing a significant Direction-by-Valence (i.e., without Group) but instead many regions showing strong (and partly regionally overlapping) main effects for Direction *and* Valence (see **Supplemental Figure 1** and **Supplemental Table 1**). In short, it can be speculated the Group-by-Direction-by-Valence interaction represents a heightened appraisal of a self-referential threat (i.e., a threat moving toward the individual) beyond more stimulus-driven responses to looming and threat information in participants previously subjected to sexual abuse.

The amygdala and rmPFC/vmPFC are not only responsive to emotional stimuli but also to social stimuli, including human faces (20, 21, 37, 38). This was seen in the current study also (see **Supplemental Table 1** and **Supplemental Figure 1**). Moreover, patients with social anxiety disorder show atypically increased activations or connectivity between the amygdala and rmPFC in response to face stimuli (39, 40). However, being previously exposed to sexual abuse was not associated with heightened responsiveness to face stimuli in the current data. These data indicate that while being previously subjected to sexual abuse may increase threat responsiveness and increase propensity for anxiety generally, it does not, at least according to these data, particularly increase responsiveness of neural responses associated with social anxiety.

The behavioral data are worthy of note. Participants were generally faster to respond to receding than looming stimuli. This presumably reflects a freeze response to approaching dangers [cf. (41)]. Interestingly, adolescents previously subjected to sexual abuse showed significantly greater increases in RT to looming relative to receding (looming-receding) stimuli relative to comparison adolescents. Notably, these group differences in increases in RT for looming relative to receding stimuli were most marked for human face stimuli. In short, these data indicate that past sexual abuse may exaggerate the acute threat response. In the context of the current study, with relatively low level threats, this manifested as increased freezing. However, with more intense threats, increased flight or even reactive aggression can be anticipated [cf. (41)]. Moreover, this may be particularly marked for the highly salient threat of approaching humans (even though a greater response to human face stimuli was not seen in the BOLD response data).

There are several caveats that should be noted with respect to the current results. First, consistent with considerable previous work (1–3), past sexual abuse was associated with significant psychiatric psychopathology (see **Table 1**). Accordingly, the current results might reflect psychopathology rather than maltreatment. Ameliorating this concern is the fact that the results of the main analysis largely held even after removal of participants with the most common psychiatric diagnoses in this sample (PTSD, MDD). Second, most adolescents who had experienced sexual abuse had also experienced other forms of maltreatment. As such, the findings might be associated with other forms of maltreatment. This possibility cannot be discounted. The current study was not designed to differentiate associations with sexual abuse relative to other forms of maltreatment. However, it is worth noting that core findings remained even when other forms of maltreatment were included as a covariate (see **Supplemental Table 7**). Third, medication rates were significantly higher for participants who had been subjected to prior sexual abuse relative to those who had not. However, the results of the main analysis held even after excluding participants on medication (stimulants, antidepressant, antipsychotics); see **Supplemental Tables 4–6**. Fourth, the face stimuli used in this study were both male and female. It is possible that face stimuli matching the sex of the perpetrator of the adolescent’s sexual abuse might exaggerate responsiveness to looming faces relative to animals.

In conclusion, we found that past sexual abuse was associated with heightened neural responsiveness to looming threats in a series of regions beyond the amygdala such as rmPFC and PCC. At the neural level, there were no group differences with respect to face stimuli or looming faces in particular. Behaviorally, participants were slower to respond to looming than receding stimuli and this effect was exaggerated in participants previously subjected to sexual abuse particularly if the looming stimulus was a human face. As such the behavioral data mirrored the BOLD response data with respect to the interaction of sexual abuse with the looming variable. However, they also suggested that there may be differential sensitization to face stimuli following sexual abuse that did not emerge in the neural data. These data are interpreted within models of rmPFC and PCC cortices that stress their role in self-referential emotional processing and emotional maintenance (18, 35, 36). Sexual abuse may lead to an exaggerated appraisal and maintenance of self-referential threats (i.e., threat, perhaps particularly human threats, moving toward the individual).

## Data Availability Statement

The datasets generated for this study are available on request to the corresponding author.

## Ethics Statement

The studies involving human participants were reviewed and approved by Boys Town National Research Hospital institutional review board. Written informed consent to participate in this study was provided by the participants’ legal guardian and written informed assent was provided by the participants.

## Author Contributions

Study PI KB conducted and is responsible for the data analysis. She had full access to all the data in the study and takes responsibility for the integrity of the data and the accuracy of the data analysis. NS, JL, JE, AE, and SV contributed to the acquisition and critical revision of the manuscript for important intellectual content. JB-L, RZ, MD, SP, and JB contributed with interpretation of the data and critical revision of the manuscript for important intellectual content.

## Funding

This research was in part supported by the National Institute of General Medical Sciences of the National Institutes of Health under award number 5P20GM109023-05 (KB) and the National Institute of Mental Health under award number K22-MH109558 (JB). SP was supported by the National Institute of Mental Health [R01MH61285] and the National Institute of Child Health and Human Development [U54 HD090256]. The funders had no role in the design and conduct of the study; collection, management, analysis, and interpretation of the data; preparation, review, or approval of the manuscript; and decision to submit the manuscript for publication.

## Conflict of Interest

The authors declare that the research was conducted in the absence of any commercial or financial relationships that could be construed as a potential conflict of interest.
