# Supplementary material for: Sexual Abuse in Adolescents Is Associated With Atypically Increased Responsiveness Within Regions Implicated in Self-Referential and Emotional Processing to Approaching Animate Threats
Source: Front Psychiatry. 2020 Jun 16;11:345. doi: 10.3389/fpsyt.2020.00345 (PMC7308525; doi:10.3389/fpsyt.2020.00345)
Supplement: Supplementary file 1 [file DataSheet_1.docx]

**SUPPLEMENTAL INFORMATION**

Supplemental Figure 1: Results for the main effects of valence, direction and type (presented at p < 0.005 to maximise overlap)

**Tables Index:**

Supplemental Table 1: Significant areas of activation from the main analysis not reported in main Table 2

Supplemental Table 2: Excluding participants with PTSD

Supplemental Table 3: Excluding participants with MDD

Supplemental Table 4: Excluding participants using stimulants

Supplemental Table 5: Excluding participants using antidepressants

Supplemental Table 6: Excluding participants using antipsychotics

Supplemental Table 7: Follow-up analyses with other maltreatment as added covariate

Supplemental Table 1. Significant areas of activation from the main group based (participants subjected to sexual abuse, comparison) analysis, not reported in main Table 2. Activations are effects observed in whole brain analyses significant at ^†^p < 0.001), corrected for multiple comparisons (significant at p < 0.05).

| **REGION** | **BA** | **Voxels** | **X** | **Y** | **Z** | **F-value** | **ηp²** |
| --- | --- | --- | --- | --- | --- | --- | --- |
|  |  |  |  |  |  |  |  |
| ***Direction*** |  |  |  |  |  |  |  |
|  |  |  |  |  |  |  |  |
| R superior frontal gyrus | 10 | 20 | 14 | 56 | 26 | 34.49 | 0.434 |
| L middle frontal gyrus | 46 | 148 | -40 | 26 | 20 | 32.23 | 0.417 |
| L middle frontal gyrus | 9/6 | 36 | -43 | 5 | 38 | 46.18 | 0.506 |
| R inferior frontal gyrus | 47 | 54 | 26 | 29 | -7 | 35.29 | 0.440 |
| R inferior frontal gyrus | 46 | 86 | 50 | 29 | 14 | 30.88 | 0.407 |
| R precentral gyrus | 6 | 30 | 41 | -4 | 32 | 48.06 | 0.516 |
| R putamen/ lentiform nucleus/ amygdala |  | 162 | 17 | 5 | 11 | 67.27 | 0.599 |
| L parahippocampal gyurs/ amygdala |  | 188 | -28 | -1 | -25 | 72.39 | 0.617 |
| L thalamus |  | 46 | -19 | -25 | -1 | 60.04 | 0.572 |
| R thalamus |  | 28 | 17 | -28 | 2 | 26.52 | 0.371 |
| R superior temporal gyrus |  | 26 | 32 | 14 | -19 | 36.60 | 0.449 |
| L/R culmen/ fusiform gyrus/ cuneus/ inferior occipital gyrus |  | 3961 | 29 | -52 | -10 | 151.19 | 0.771 |
|  |  |  |  |  |  |  |  |
| ***Type*** |  |  |  |  |  |  |  |
|  |  |  |  |  |  |  |  |
| R medial frontal gyrus | 10 | 65 | 5 | 44 | -7 | 26.41 | 0.370 |
| R precunues | 31 | 64 | 5 | -58 | 32 | 36.66 | 0.449 |
| L thalamus |  | 23 | -16 | -28 | -1 | 35.51 | 0.441 |
| L/R culmen/ fusiform gyrus/ cuneus/ inferior occipital gyrus |  | 6132 | 26 | -31 | -19 | 165.87 | 0.787 |
|  |  |  |  |  |  |  |  |
| ***Valence*** |  |  |  |  |  |  |  |
|  |  |  |  |  |  |  |  |
| R superior frontal gyrus | 6/8 | 108 | 11 | 20 | 56 | 24.27 | 0.350 |
| L middle frontal gyrus | 10 | 40 | -34 | 44 | 8 | 23.58 | 0.344 |
| R middle frontal gyrus | 10 | 21 | 44 | 47 | 5 | 24.77 | 0.355 |
| R middle frontal gyrus | 6 | 25 | 35 | 11 | 50 | 22.21 | 0.330 |
| L middle frontal gyrus |  | 78 | -16 | 2 | 62 | 33.61 | 0.428 |
| R lingual gyrus | 19 | 21 | 20 | -49 | -1 | 24.61 | 0.353 |
| R cuneus | 23 | 618 | 14 | -70 | 11 | 27.87 | 0.381 |
|  |  |  |  |  |  |  |  |
| ***Direction-by-Type*** |  |  |  |  |  |  |  |
|  |  |  |  |  |  |  |  |
| R middle occipital gyrus | 19 | 26 | 38 | -70 | 11 | 20.22 | 0.310 |
| R cuneus | 17 | 26 | 8 | -79 | 8 | 29.26 | 0.394 |
|  |  |  |  |  |  |  |  |
| ***Type-by-Valence*** |  |  |  |  |  |  |  |
|  |  |  |  |  |  |  |  |
| L fusiform gyrus | 19 | 26 | -31 | -82 | -13 | 22.33 | 0.332 |

Supplemental Table 2. Significant areas of activation from the analysis exluding participants wth a diagnosis of PTSD. Activations are from whole brain analyses significant at p < 0.005, corrected for multiple comparisons significant at p < 0.05.

| **REGION** | **BA** | **Voxels** | **X** | **Y** | **Z** | **F-value** |
| --- | --- | --- | --- | --- | --- | --- |
|  |  |  |  |  |  |  |
| ***Group-by-Direction-by-Valence*** |  |  |  |  |  |  |
| L/R medial frontal gyrus | 10 | 167 | 2 | 56 | 5 | 28.05 |
| L superior frontal gyrus | 10 | 42 | -22 | 53 | 26 | 17.70 |
| L superior frontal gyrus | 8 | 61 | -11 | 44 | 53 | 14.48 |
| R posterior cingulate cortex | 31 | 126 | 5 | -43 | 11 | 17.17 |
| R superior temporal gyrus | 38 | 53 | 41 | 14 | -31 | 20.75 |
| R inferior temporal gyrus | 20 | 29 | 50 | -7 | -19 | 19.76 |

Supplemental Table 3. Significant areas of activation from the analysis exluding participants wth a diagnosis of MDD. Activations are from whole brain analyses significant at p< 0.005, corrected for multiple comparisons significant at p < 0.05.

| **REGION** | **BA** | **Voxels** | **X** | **Y** | **Z** | **F-value** |
| --- | --- | --- | --- | --- | --- | --- |
|  |  |  |  |  |  |  |
| ***Group-by-Direction-by-Valence*** |  |  |  |  |  |  |
| L medial frontal gyrus/ superior frontal gyrus | 10 | 294 | -1 | 56 | 5 | 21.56 |
| L superior frontal gyrus | 8 | 52 | -11 | 29 | 53 | 16.13 |
| L posterior cingulate cortex | 31 | 196 | -4 | -49 | 32 | 18.48 |
| R superior temporal gyrus | 38 | 93 | 41 | 14 | -31 | 24.73 |
| R inferior temporal gyrus | 20 | 37 | 50 | -7 | -19 | 21.48 |

Supplemental Table 4. Stimulants use as an added covariate. Significant areas of activation from the 2 (Group: Exposed to sexual abuse, Comparison) by 2 (Direction: Looming, Receding) by 2 (Type: Human, Animal) by 2 (Valence: Threatening, Neutral) ANCOVA with use of stimulants (ON, OFF) as covariate. Activations are effects observed in whole brain analyses significant at p < 0.001, corrected for multiple comparisons (significant at p < 0.05).

| **REGION** | **BA** | **Voxels** | **X** | **Y** | **Z** | **F-value** | **ηp²** |
| --- | --- | --- | --- | --- | --- | --- | --- |
|  |  |  |  |  |  |  |  |
| ***Group-by-Direction-by-Valence*** |  |  |  |  |  |  |  |
| R medial frontal gyrus/ ACC | 10/ 24 | 175 | 5 | 35 | -1 | 21.42 | .327 |
| L superior frontal gyrus | 8 | 26 | -13 | 44 | 41 | 18.90 | .300 |
| L posterior cingulate cortex | 31 | 75 | -1 | -46 | 29 | 17.73 | .287 |
| R superior temporal gyrus^005 | 38 | 71 | 44 | 14 | -34 | 26.52 | .376 |
| R inferior temporal gyrus | 20 | 21 | 50 | -7 | -19 | 30.86 | .412 |
| L middle frontal gyrus | 11 | 24 | -28 | 41 | -4 | 20.29 | .316 |

Supplemental Table 5. Antidepressants use as an added covariate. Significant areas of activation from the 2 (Group: Exposed to sexual abuse, Comparison) by 2 (Direction: Looming, Receding) by 2 (Type: Human, Animal) by 2 (Valence: Threatening, Neutral) ANCOVA with use of antidepressants (ON, OFF) as covariate. Activations are effects observed in whole brain analyses significant at p < 0.005, corrected for multiple comparisons (significant at p < 0.05).

| **REGION** | **BA** | **Voxels** | **X** | **Y** | **Z** | **F-value** | **ηp²** |
| --- | --- | --- | --- | --- | --- | --- | --- |
|  |  |  |  |  |  |  |  |
| ***Group-by-Direction-by-Valence*** |  |  |  |  |  |  |  |
| R medial frontal gyrus | 10 | 56 | 2 | 59 | 17 | 31.75 | .419 |
| L superior frontal gyrus^ | 8/6 | 26 | -10 | 29 | 53 | 20.76 | .321 |
| L superior frontal gyrus | 10 | 38 | -22 | 53 | 26 | 23.14 | .345 |
| L posterior cingulate cortex | 31 | 24 | -1 | -49 | 29 | 27.53 | .385 |
| R superior temporal gyrus^ | 38 | 51 | 44 | 14 | -34 | 26.43 | .376 |
| R inferior temporal gyrus^ | 20 | 33 | 53 | -7 | -19 | 20.27 | .315 |

Supplemental Table 6. Antipsychotics use as an added covariate. Significant areas of activation from the 2 (Group: Exposed to sexual abuse, Comparison) by 2 (Direction: Looming, Receding) by 2 (Type: Human, Animal) by 2 (Valence: Threatening, Neutral) ANCOVA with use of antipsychoatics (ON, OFF) as covariate. Activations are effects observed in whole brain analyses significant at p < 0.005, corrected for multiple comparisons (significant at p < 0.05).

| **REGION** | **BA** | **Voxels** | **X** | **Y** | **Z** | **F-value** | **ηp²** |
| --- | --- | --- | --- | --- | --- | --- | --- |
|  |  |  |  |  |  |  |  |
| ***Group-by-Direction-by-Valence*** |  |  |  |  |  |  |  |
| R medial frontal gyrus/ ACC | 10/ 24 | 156 | 5 | 35 | -1 | 28.22 | .391 |
| L superior frontal gyrus^ | 8/6 | 67 | -10 | 29 | 53 | 25.79 | .370 |
| L posterior cingulate cortex | 65 | 24 | -1 | -22 | 38 | 17.49 | .284 |
| R superior temporal gyrus | 38 | 21 | 44 | 14 | -34 | 38.51 | .467 |
| R inferior temporal gyrus^ | 20 | 38 | 50 | -7 | -19 | 21.27 | .344 |

Supplemental Table 7. Significant areas of activation from the 2 (Direction: Looming, Receding) by 2 (Type: Human, Animal) by 2 (Valence: Threatening, Neutral) ANCOVA with the participants’ Blom transformed CTQ other maltreatment (emotional abuse, physical abuse, emotional neglect, physical neglect) scores as a added continuous variate. Activations are from whole brain analyses significant at p < 0.005, significant at p < 0.05.

| **REGION** | **BA** | **Voxels** | **X** | **Y** | **Z** | **F-value** | **ηp²** |
| --- | --- | --- | --- | --- | --- | --- | --- |
|  |  |  |  |  |  |  |  |
| ***CTQ sexual abuse scores-by-Direction-by-Valence*** | | | | | | | |
| L medial frontal gyrus | 10/9 | 25 | -1 | 56 | 26 | 20.66 | .319 |
| L superior frontal gyrus | 8 | 31 | -10 | 29 | 50 | 28.47 | .393 |
| R inferior frontal gyrus | 20 | 20 | 53 | -7 | -19 | 15.32 | .258 |

**FIGURE LEGENDS**

Supplemental Figure 1. Main effects of Valence, Direction and Type Task illustration.

**REFERENCE LIST**

1. Wechsler D (2011): *Wechsler Abbreviated Scale of Intelligence–Second Edition (WASI-II)*. San Antonio, TX: NCS Pearson.

2. Blair KS, Smith BW, Mitchell DG, Morton J, Vythilingam M, Pessoa L*, et al.* (2007): Modulation of emotion by cognition and cognition by emotion*.* *Neuroimage* 35(1): 430-40.

3. Hwang S, Nolan ZT, White SF, Williams WC, Sinclair S, and Blair RJ (2016): Dual neurocircuitry dysfunctions in disruptive behavior disorders: emotional responding and response inhibition*.* *Psychological Medicine* 46(7): 1485-96.

4. Lang PJ and Greenwald MK (1988): *The international affective picture system standardization procedure and initial group results for affective judgements: Technical reports 1A & 1B*. Gainesville: Center for Research in Psychophysiology, University of Florida.

5. Birmaher B, Brent DA, Chiappetta L, Bridge J, Monga S, and Baugher M (1999): Psychometric properties of the Screen for Child Anxiety Related Emotional Disorders (SCARED): a replication study*.* *J Am Acad Child Adolesc Psychiatry* 38(10): 1230-6.

6. Angold A, Costello EJ, Messer SC, and Pickles A (1995): Development of a short questionnaire for use in epidemiological studies of depression in children and adolescents*.* *Int J Methods Psychiatr Res* 5(4): 237-249.

7. Cox RW (1996): AFNI: software for analysis and visualization of functional magnetic resonance neuroimages*.* *Computers and Biomedical Research* 29: 162-73.

8. Talairach J and Tournoux P (1988): *Co-planar stereotaxic atlas of the human brain*. Stuttgart: Thieme.

9. Blom G (1958): *Statistical Estimates and Transformed Beta-Variables*. John Wiley & Sons.

10. Steiger JH (1980): Tests for comparing elements of a correlation matrix*.* *Psychological Bulletin* 87: 245–251.

11. Blair KS, Vythilingam M, Crowe SL, McCaffrey DE, Ng P, Wu CC*, et al.* (2012): Cognitive control of attention is differentially affected in trauma-exposed individuals with and without post-traumatic stress disorder*.* *Psychol Med*: 1-11.

12. Blair KS, Geraci M, Smith BW, Hollon N, Devido J, Otero M*, et al.* (2012): Reduced dorsal anterior cingulate cortical activity during emotional regulation and top-down attentional control in generalized social phobia, generalized anxiety disorder, and comorbid generalized social phobia/generalized anxiety disorder*.* *Biol Psychiatry* 72(6): 476-82.

13. Blair KS, Otero M, Teng C, Geraci M, Lewis E, Hollon N*, et al.* (2016): Learning from other people's fear: amygdala-based social reference learning in social anxiety disorder*.* *Psychol Med* 46(14): 2943-2953.

14. Etkin A and Wager TD (2007): Functional Neuroimaging of Anxiety: A Meta-Analysis of Emotional Processing in PTSD, Social Anxiety Disorder, and Specific Phobia*.* *Am J Psychiatry* 164(10): 1476-88.

15. Williams LE, Oler JA, Fox AS, McFarlin DR, Rogers GM, Jesson MA*, et al.* (2015): Fear of the unknown: uncertain anticipation reveals amygdala alterations in childhood anxiety disorders*.* *Neuropsychopharmacology* 40(6): 1428-35.
