# Supplementary figures and images for: Sexual Abuse in Adolescents Is Associated With Atypically Increased Responsiveness Within Regions Implicated in Self-Referential and Emotional Processing to Approaching Animate Threats
Source: Front Psychiatry. 2020 Jun 16;11:345. doi: 10.3389/fpsyt.2020.00345 (PMC7308525; doi:10.3389/fpsyt.2020.00345)

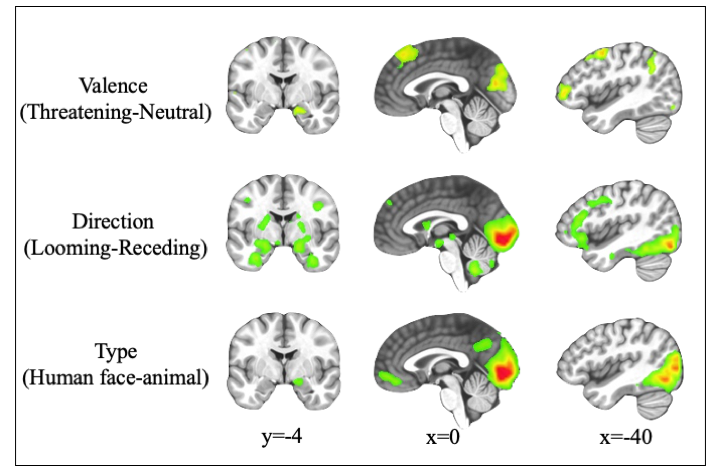

Supplement: Supplementary file 2 [file Image_1.tiff]
